# Dopaminergic treatment of patients with acromegaly: still kicking after all these years

**DOI:** 10.20945/2359-3997000000502

**Published:** 2022-07-01

**Affiliations:** 1 Universidade Federal de São Paulo Escola Paulista de Medicina Divisão de Endocrinologia e Metabolismo Brazil Unidade de Neuroendocrinologia, Divisão de Endocrinologia e Metabolismo, Escola Paulista de Medicina, Universidade Federal de São Paulo (EPM-Unifesp)

In this issue of the *Archives of Endocrinology and Metabolism*, the article “Efficacy of cabergoline add-on therapy in patients with acromegaly resistance to somatostatin analogs treatment and the review of literature”, by Kizilgul and cols. (1), shares an excellent opportunity to review the history and to reappraise the role of dopaminergic treatment in acromegaly.

It all started 50 years ago, when Liuzzi and cols. (2) reported in *The Journal of Clinical Endocrinology and Metabolism* an inhibitory effect of L-Dopa in patients with acromegaly. That observation was particularly intriguing because L-Dopa, a dopamine precursor, was known to stimulate GH secretion in healthy subjects (3). Here is a quote from the discussion in that seminal paper: *On the basis of clinical and experimental knowledge we are unable to explain these results.... we believe that the paradoxical fall of plasma GH we observed in some patients affected by acromegaly is of interest and needs more extensive studies. It suggests a new therapeutic approach to the treatment of acromegaly*.

Indeed, in the following years, that same group of investigators showed that a new ergot derivative, 2-Br-alpha-ergocryptine (bromocriptine), a dopamine agonist, was also able to reduce growth hormone secretion in patients with acromegaly (4). Soon after, bromocriptine proved to be even more effective in reducing prolactin secretion in various physiological and pathological conditions and became the first choice treatment for prolactinomas (5). The effect of bromocriptine on growth hormone secretion and tumor growth in acromegaly was later shown to be mediated by dopaminergic receptors (particularly type 2), which are usually expressed in growth hormone and other pituitary adenomas (6). Notwithstanding its modest efficacy and several side effects, bromocriptine remained, for a whole decade, as the only pharmacological treatment available for acromegaly.

In the mid-eighties, treatment of acromegaly changed dramatically with the development of octreotide, a somatostatin analogue much more effective than bromocriptine in controlling growth hormone secretion and tumor growth in patients with acromegaly (7). Thereafter, octreotide and, also, lanreotide (another first generation somatostatin analog), became the first line of pharmacological treatment for that disease. Nevertheless, almost half of patients do not attain disease control under treatment with those somatostatin analogs even with higher than usual doses (8).

At about the same time, cabergoline, another ergot-derivative with a more potent dopaminergic activity and less side effects than bromocriptine, showed a remarkable superiority in relation to bromocriptine in the treatment of prolactinomas (9). Later, in the late 90’s, two preliminary reports of treatment of acromegaly with cabergoline, alone or in combination with somatostatin analogues, showed promising outcomes (10,11). Those results were confirmed and further expanded by several investigators in the following years, but the potential value of cabergoline in acromegaly was overshadowed by the advent of first-generation somatostatin analogs (12-15). Besides, marketing authorization has never been sought for cabergoline in this indication and, thus, cabergoline remains an off-label drug in acromegaly.

In the new millennium, along with various trials on cabergoline treatment of acromegaly, novel scientific developments brought two new molecules for acromegaly: pegvisomant, the first GH receptor blocker that decreases the peripheral effects of GH, and pasireotide, a second generation somatostatin analog with extended affinity for somatostatin receptor subtypes besides the subtype 2 receptor (16,17). Both drugs, through completely different mechanisms and under proper indications, have independently improved the control of acromegaly in patients not controlled by first generation somatostatin analogs although at a high financial cost and, in the case of pasireotide, with frequent undesirable side effects in glycemic control (18).

Back to the present, the study by Kizilgul and cols. (1), although it is retrospective, corroborates the overall efficacy of adding cabergoline to the treatment of patients resistant to first generation analogs, as well as the even higher efficacy of cabergoline in patients with mild/moderate elevations in IGF-1 levels (IGF-1<2.5 ULN). In this regard, I would like to bring up a frequently ignored practical observation: in many patients successfully responding to the addition of cabergoline to somatostatin analogs, cabergoline alone is able to sustain disease control after the interruption of the somatostatin analog (12,13). This is more likely to occur in those patients whose initial response to the somatostatin analog was modest or who had no response at all. Thus, either as an add-on therapy or as a monotherapy, cabergoline can improve acromegaly control with reduced treatment burden for the patient and lesser financial cost for the public health system.

Last, but not least, going through the literature review in Kizilgul´s article, one cannot overlook the significant contribution of several research groups from Brazil (12-15). As a matter of fact, the largest part of all published prospective studies on that theme came from our country and the amount of patients included represents more than 80% of the total subjects in such trials. That is not only remarkable, but even more so when considering that those studies were originated from the initiative of our investigators without any kind of Pharma support.

To sum up, the publication by Kizilgul and cols. (1) showing once more the efficacy of cabergoline in the treatment of acromegaly is a timely reminder that dopaminergic treatment still has an important role, at a relatively low cost, in the current management of acromegaly. It is noteworthy that cabergoline is included in the drugs’ list for the treatment of acromegaly in our public health system.
